# Structural Brain Correlates of Insufficient Sleep in Adolescents: A Narrative Review of MRI Evidence

**DOI:** 10.3390/children13070903

**Published:** 2026-07-08

**Authors:** Rishi Ananth, Justyna Swierz, Bella Shafizadeh, Shenée Martin, Miranda M. Lim, Yeilim Cho

**Affiliations:** 1 Department of Psychiatry and Behavioral Sciences, University of Washington, Seattle, WA 98195, USA; rka8@uw.edu (R.A.); ids8943@uw.edu (B.S.); shencm@uw.edu (S.M.); 2 VISN 20 Mental Illness Research, Education and Clinical Center, Seattle, WA 98108, USA; swierzju@uw.edu; 3 Department of Psychiatry and Behavioral Sciences, University of Washington School of Medicine, Seattle, WA 98195, USA; 4 VISN 20 Northwest Mental Illness Research, Education and Clinical Center (MIRECC), VA Portland Health Care System, Portland, OR 97239, USA; lmir@ohsu.edu; 5 Neurology Service, Research Service, VA Portland Health Care System, Portland, OR 97239, USA; 6 Department of Neurology, Oregon Alzheimer’s Disease Research Center, Oregon Health & Science University, Portland, OR 97239, USA

**Keywords:** insufficient sleep, adolescents, structural neuroimaging, MRI, brain development, diffusion tensor imaging, cortical thickness

## Abstract

Insufficient sleep represents a public health challenge affecting the majority of adolescents in industrialized societies, with fewer than 30% of high school students obtaining the recommended 8–10 h of nightly sleep. Chronic effects of insufficient sleep have been shown to affect a myriad of functions including brain development, metabolism, mental health, school performance, and social relationships. While these biological and functional consequences of adolescent insufficient sleep have been addressed in prior reviews, no review has systematically examined the effects of insufficient sleep on structural brain development. Herein, we address this gap by reviewing the growing magnetic resonance imaging (MRI) literature linking adolescent sleep patterns to brain morphology. This review systematically synthesizes study findings from MRI sequences spanning diffusion tensor imaging (DTI), voxel-based morphometry, and structural volumetrics, including gray matter, white matter, and cortical thickness as it relates to sleep duration, sleep regularity, and sleep quality. In summary, results from these studies link insufficient sleep to measurable alterations in white matter microstructure, cortical thickness, and gray matter volume during adolescence, establishing detectable effects of brain morphology during a period of heightened neurobiological vulnerability. Protecting adolescent sleep represents an investment in both immediate well-being and long-term health outcomes extending into adulthood.

## 1. Introduction

Insufficient sleep presents as a highly prevalent yet underrecognized public health challenge facing adolescent populations (ages 10–19). The International Classification of Disease, 10th Revision (ICD-10), recognizes insufficient sleep as a non-organic sleep disorder (code G47.8) characterized by a persistent pattern of sleep loss due to reduced sleep opportunity, resulting in excessive daytime sleepiness or other symptoms that impair functioning [1]. It is important to distinguish the focus of this review from related but distinct clinical constructs. Behavioral sleep insufficiency refers to chronically curtailed sleep opportunity arising from circadian-environmental mismatch. This differs from insomnia disorder, which is characterized by difficulty initiating or maintaining sleep despite adequate opportunity, and from formal insufficient sleep syndrome, a clinical diagnosis requiring documented sleep curtailment with daytime consequences.

The adolescent period represents an important window of neurodevelopmental change during which synaptic reorganization and brain maturation are actively ongoing. Longitudinal neuroimaging studies demonstrate that cortical gray matter follows an inverted U-shaped developmental trajectory with regional specificity, peaking at approximately age 12 in frontal and parietal cortices and around age 16 in the temporal lobe [2]. Concurrent with gray matter changes, white matter volume increases linearly throughout adolescence and into early adulthood, reflecting ongoing myelination that supports faster and more efficient neural transmission. Notably, this myelination process is dependent on sleep, and REM sleep in particular, linking sleep duration during adolescence to a critical axis of structural brain maturation [3]. Postmortem analyses further indicate that adolescence is marked by substantial synaptic pruning, with region-specific reductions in synaptic density extending beyond early childhood [4]. Together, these changes underscore why adolescence constitutes a uniquely sensitive period in which the brain is both highly plastic and particularly vulnerable to disruptions that may have lasting neurodevelopmental consequences. Yet, fewer than 30% of U.S. high school students obtain the recommended 8–10 h of nightly sleep [5], and sleep restriction during this period has been associated with impairments in sustained attention, executive function, and academic achievement [6,7,8,9].

Several prior reviews have examined aspects of adolescent sleep and brain development, covering sleep physiology, cognition, and mental health [10,11,12,13,14,15,16]. However, no prior review has synthesized the growing structural neuroimaging literature, including MRI structural volumetrics, cortical thickness, and voxel-based morphometry studies, linking adolescent sleep patterns to brain morphology. Moreover, several large-scale neuroimaging investigations published since 2021, including findings from the Adolescent Brain Cognitive Development (ABCD) Study [17], have substantially expanded the evidence beyond what was available to earlier reviews. The present review addresses this gap by first summarizing the biological, environmental, and functional context of adolescent insufficient sleep, and then providing a focused synthesis of structural MRI evidence linking insufficient sleep to brain morphology during adolescence. This integrative framework positions insufficient sleep not only as a behavioral pattern but as a condition with potential neuroanatomical associations during a period of heightened developmental vulnerability.

## 2. Methods

### 2.1. Literature Search Strategy

This narrative review synthesizes evidence across five domains of adolescent insufficient sleep: circadian biology, homeostatic sleep regulation, environmental contributors, cognitive consequences, and structural neuroimaging. A systematic review methodology was not pursued, as the primary aim of this review is the conceptual synthesis and the mechanistic framework building across domains, which is better suited to a narrative methodology than to the quantitative collection that is prioritized by systematic reviews.

We conducted targeted literature searches of PubMed, Web of Science, and PsycINFO through December 2025 for each domain. Domain-specific search terms included combinations of: (“adolescent” OR “teenager” OR “youth”) AND (“insufficient sleep” OR “sleep deprivation” OR “sleep restriction”) combined with domain-relevant terms including (“circadian” OR “melatonin” OR “chronotype”), (“school start time” OR “screen time” OR “electronic devices”), (“cognition” OR “academic performance” OR “executive function”), and (“MRI” OR “neuroimaging” OR “cortical thickness” OR “white matter” OR “gray matter volume”). Searches were supplemented by manual review of reference lists of included articles.

We prioritized peer-reviewed, English-language studies in human adolescent populations (ages 10–19), with emphasis on meta-analyses, experimental studies, and studies employing objective sleep measurement. Studies published in languages other than English, non-peer-reviewed sources, and studies in which adolescent-specific data is not isolated from adult samples were excluded. For the structural neuroimaging section specifically, adult studies were included selectively where adolescent literature was limited, in order to provide developmental context; relevance to adolescent neurodevelopment is stated explicitly where such studies are discussed. A total of 43 studies were included in this narrative synthesis.

### 2.2. Evidence Evaluation

We distinguish between: (1) Experimental vs. observational evidence; (2) Animal model vs. human data, (3) Cross-sectional vs. longitudinal studies, (4) Sample sizes and study quality considerations.

### 2.3. Figure Generation

**Figure 1:**
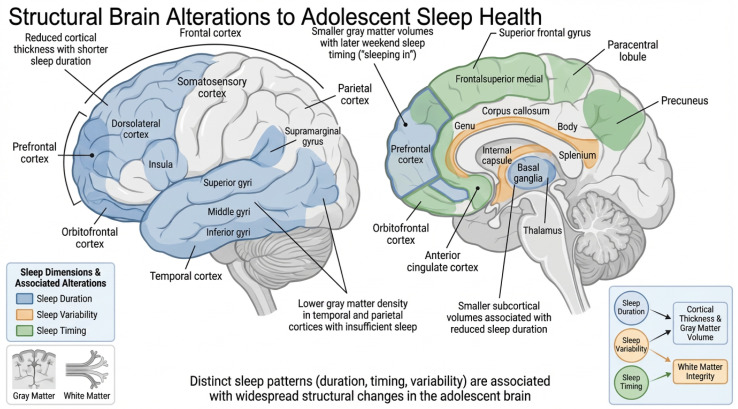
Structural brain regions associated with distinct sleep dimensions in adolescents. Brain areas implicated in neuroimaging studies are color-coded by sleep dimension: sleep duration (blue), sleep variability (green), and sleep timing (yellow). Regions depicted reflect associations reported in the studies reviewed; many findings derive from single cross-sectional studies and await replication.

Figure 1 was generated using FigureLabs AI tool (Nano Banana Pro, accessed 29 January 2026) and manual edits. The authors provided detailed specifications for each panel, including specific text content, visual elements, layout structure, and scientific content to be depicted. All scientific concepts, terminology, and information presented in the figure were author-specified and are supported by the cited literature.

## 3. Background: Biological, Environmental, and Functional Context of Adolescent Insufficient Sleep

The biological, environmental, and functional dimensions of adolescent insufficient sleep have been examined extensively in prior reviews [10,11,12,13,14,15,16]. Here we provide a brief summary of these domains to establish the context for the structural neuroimaging evidence reviewed in the following section.

### 3.1. Circadian Phase Delay and Homeostatic Sleep Regulation

Circadian phase delay represents a fundamental biological contributor to adolescent insufficient sleep. Across adolescence, the circadian timing system undergoes progressive delays toward later sleep-wake patterns, correlating with pubertal maturation [18,19]. This produces an “evening chronotype” that increasingly conflicts with early school start times [20]. As adolescents are required to wake during their biological night, both circadian drive for wakefulness and homeostatic sleep pressure remain incomplete, resulting in chronic sleep restriction that sets the stage for the structural and functional outcomes discussed below [21,22].

### 3.2. Environmental Contributors: School Start Times and Screen Exposure

Early school start times represent a major modifiable contributor to adolescent insufficient sleep; despite American Academy of Pediatrics recommendations for start times no earlier than 8:30 AM, most U.S. middle and high schools begin earlier [23,24]. Studies of delayed school start times consistently demonstrate increased sleep duration, with one large cohort reporting approximately 43 additional minutes of nightly sleep following implementation [25]. Electronic device use compounds this problem: evening exposure to short-wavelength light from screens suppresses melatonin and delays sleep onset [26,27], while devices in the bedroom are independently associated with shorter sleep duration [28,29].

### 3.3. Functional Consequences: Cognitive Performance and Academic Achievement

Sleep duration and regularity are associated with academic outcomes in adolescents, with shorter and more irregular sleep linked to poorer school performance [6]. Insufficient sleep impairs sustained attention and executive function [7] with the prefrontal cortex, which undergoes longer development through adolescence, showing particular vulnerability [8,9]. These cognitive and academic associations provide important functional context for the structural findings reviewed below, particularly regarding the medial prefrontal cortex.

An important remaining question is whether chronically insufficient or irregular sleep is also associated with detectable differences in the structure of the developing brain. The following section addresses this question through a focused review of structural MRI evidence.

## 4. Structural Brain Correlates of Insufficient Sleep in Adolescents: MRI Evidence

MRI provides non-invasive quantification of brain structure in living adolescents, enabling investigation of whether sleep patterns relate to ongoing neurodevelopmental processes [2,30]. Unlike functional measures that reflect transient cognitive states, structural MRI measures such as cortical thickness, gray matter volume, and white matter integrity offer relatively stable markers of developmental trajectories that may be sensitive to chronic environmental factors including sleep patterns [3,31]. While the findings reviewed below are associative and cannot establish causality, they provide important insights into the relationship between sleep patterns and neurodevelopmental trajectories. Table 1 summarizes the study designs, samples, and principal findings discussed in this section, and Figure 1 provides a corresponding anatomical summary of the regions implicated across sleep dimensions as per the studies.

Adult and young adult studies are included selectively, where adolescent-specific literature remains limited, to provide developmental context rather than direct evidence. The relevance and limits of each comparison are noted at the point of discussion.

### 4.1. Sleep Duration

Large-scale structural neuroimaging studies demonstrate associations between sleep duration and cortical structure. In a cross-sectional analysis of the National Adolescent and Parent Sleep databank (N = 225 participants aged 9–25 years), shorter sleep duration was associated with reduced cortical thickness in frontal, parietal, and temporal regions [31]. Effects extended beyond the cortex to subcortical structures including the basal ganglia and thalamus [31,32]. The widespread nature of these structural correlations suggests that insufficient sleep may have global rather than region-specific effects on brain maturation.

These findings somewhat parallel what is seen in adults, though the adult literature is more often longitudinal and shows less consistent results. A longitudinal study of 122 cognitively normal older adults from the Baltimore Longitudinal Study of Aging, followed with serial MRI over 8 years, found that both short and long sleep durations were associated with accelerated cortical thinning in frontal and temporal regions [33]; given the substantial age and developmental difference from adolescence, this finding is best interpreted as evidence that sleep-duration sensitivity persists across the lifespan rather than as a direct model of adolescent neurodevelopment. A separate longitudinal cohort tracking self-reported sleep duration trajectories over 28 years in 613 adults reported no differences in gray matter volume, white matter microstructure, or cognition across four sleep duration trajectory groups [34]. These null findings suggest that the structural sensitivity to sleep duration observed during adolescent neurodevelopment, a period of heightened plasticity, may not be as consistently detectable in the structurally mature adult brain.

### 4.2. Sleep Regularity

Cross-sectional evidence from an adolescent cohort (N = 177, mean age 14 years) demonstrates that sleep timing patterns, particularly during weekends, show associations with regional gray matter volumes [35]. Using voxel-based morphometry, investigators found that later weekend bedtimes correlated with smaller gray matter volumes in three distinct areas: the right precuneus and paracentral lobule, the right middle/superior frontal gyrus, and the right frontal superior medial cortex extending into left anterior cingulate cortex [35]. Importantly, later weekend wake-up times were associated with reduced gray matter volumes in the left medial orbitofrontal cortex and left anterior cingulate cortex [35]. These findings suggest that sleep timing irregularities, particularly the phenomenon of “sleeping in” on weekends as a compensatory strategy for weekday sleep debt, may have distinct structural brain correlates beyond those associated with total sleep duration alone.

The medial prefrontal cortex emerges as a particularly vulnerable brain region to variations in adolescent sleep patterns, with implications for both structural development and academic performance. In the previously mentioned cross-sectional cohort, medial prefrontal cortex gray matter volume correlated with both weekend bedtime and weekend wake-up time, and was additionally associated with school grade average [35]. Mediation analyses revealed that weekend bedtime accounted for 43.8% of the total effect between gray matter volume in the frontal superior medial cortex and anterior cingulate cortex and school performance (*p* < 0.001) [35]. This convergence of structural, sleep, and performance measures in medial prefrontal regions suggests that this brain area may represent a nexus where insufficient sleep impacts both neurodevelopmental trajectories and functional outcomes during adolescence; as this evidence is cross-sectional, however, it cannot establish whether sleep timing drives these structural differences or vice versa.

### 4.3. Sleep Quality

Cross-sectional voxel-based morphometry studies demonstrate that poorer sleep quality is associated with reduced gray matter volume across multiple functionally significant cortical regions in adolescents, including the orbitofrontal cortex, insula, and frontal brain networks involved in higher-order cognitive and affective processing [36]. Importantly, the severity of sleep disturbance correlated with the magnitude of neural volume reduction, suggesting dose-response relationships [36]. These regional effects align with the cognitive and emotional deficits observed in sleep-deprived adolescents, providing structural correlates for functional impairments.

Adult neuroimaging studies offer convergent, though not directly generalizable, support for these regional associations. A longitudinal study of 147 adults (mean 53.9 ± 15.5 years) found that poor sleep quality was associated with reduced frontal cortical volume cross-sectionally and with faster atrophy across widespread frontal, temporal, and parietal regions over a mean follow-up time of 3.5 years [37]; because this sample is substantially older and the underlying biology involves age-related atrophy rather than adolescent maturation, the finding is best read as evidence that frontal regions remain sleep-sensitive across the lifespan, not as a model for adolescent development. Similarly, a cross-sectional study of 1,683 cognitively unimpaired middle-aged adults found that those with insomnia displayed lower gray matter volume in the orbitofrontal cortex, precuneus, posterior cingulate cortex, and thalamus [38], a regional pattern that closely mirrors the orbitofrontal and frontal effects shown in adolescents despite the different developmental context. One aspect that remains unexplored in the adolescent literature is sex specificity. A cross-sectional voxel-based morphometry study of 1074 young adults (ages 22–35) from the Human Connectome Project found that poor sleep quality was associated with reduced gray matter volume in the parahippocampal gyrus and right hippocampus in females but not males [39]; as the closest age group to late adolescence among the studies discussed here, this raises the question of whether similar sex-dependent effects exist but remain unexplored in adolescent populations specifically.

### 4.4. White Matter Microstructure

Diffusion tensor imaging studies provide evidence that sleep patterns influence white matter microstructural development during adolescence. A longitudinal investigation measuring sleep 1.5 years prior to neuroimaging in adolescents aged 13–19 years found that sleep variability (night-to-night inconsistency) predicts reduced fractional anisotropy in major white matter tracts, independent of average sleep duration [3]. Specific effects were documented across association tracts, projection tracts, and the interhemispheric tract, encompassing regions such as the superior longitudinal fasciculus, internal capsule, and corpus callosum [3], suggesting that consistent sleep schedules may be as important as adequate duration for optimal white matter development. This finding has practical implications for adolescent sleep interventions, which have traditionally focused on increasing total sleep time but may need to equally emphasize sleep schedule regularity.

Cross-sectional findings in adult and young adult populations agree, although the developmental relevance of each comparison varies. In a DTI study of 103 healthy young (aged 18–30) and older (aged 60–90) adults, greater rest-activity rhythm stability measured via seven days of actigraphy was associated with higher fractional anisotropy primarily in the corpus callosum and anterior corona radiata, with this relationship not moderated by the age group [40]. It should be noted that the inclusion of a young-adult subgroup close in age to late adolescence strengthens the relevance of this comparison. The convergence on the corpus callosum in both the adolescent sleep variability data and this adult rest-activity rhythm study suggests that white matter connectivity might be especially sensitive to sleep-wake regularity across the lifespan. Additional cross-sectional DTI evidence in young adults confirms the sensitivity of overlapping white matter tracts to sleep parameters: in a sample of 33 young adults (aged 23–29), shorter actigraphy-measured sleep duration was associated with reduced fractional anisotropy in the orbitofrontal region and superior corona radiata [41], and in a separate sample of 73 young adults (22.5 ± 0.2 years), long sleep duration was associated with reduced fractional anisotropy and increased radial diffusivity in the corona radiata, corpus callosum, superior longitudinal fasciculus, and anterior thalamic radiation [42]. The involvement of the superior longitudinal fasciculus and corpus callosum in both the adolescent and young-adult samples, the latter being closest in developmental stage to late adolescence, supports the view that these tracts are sensitive to sleep parameters across the transition from adolescence into early adulthood.

### 4.5. Population-Level Evidence from the ABCD Study

The largest pediatric neuroimaging investigation to date, a cross-sectional analysis utilizing the Adolescent Brain Cognitive Development (ABCD) Study dataset (N = 11,067 children aged 9–11 years), provides population-level associations between sleep duration, brain structure, and psychopathology [17]. Shorter sleep duration is associated with smaller volumes in neural regions such as the orbitofrontal cortex, prefrontal cortex, temporal cortex, precuneus, and supramarginal gyrus [17]. These structural differences were accompanied by higher psychopathology scores related to depression, anxiety, and impulsivity. Mediation analyses revealed that brain volume partially mediates relationships between sleep duration and behavioral outcomes, suggesting that structural brain alterations represent one mechanistic pathway through which insufficient sleep influences mental health and behavior [17]. Given its scale, this dataset offers the strongest currently available basis for generalizing structural associations to the broader adolescent population, though as a cross-sectional design it remains subject to the same causal limitations as the smaller studies discussed above.

### 4.6. Confounding and Unmeasured Factors

The studies summarized above generally did not adjust uniformly for factors that may independently influence both sleep and brain structure. Socioeconomic status, mental health comorbidities (including depression and anxiety), physical activity, obesity, and broader environmental exposures have each been linked separately to structural brain differences in pediatric and adult populations, and could plausibly confound or mediate the associations reported above. The degree to which individual studies accounted for these factors varied, and most analyses summarized in Table 1 did not report adjustment for the full set of these variables.

### 4.7. Summary of Structural Neuroimaging Evidence

Taken together, these findings converge on a consistent picture: insufficient sleep during adolescence is associated with measurable differences in brain structure across multiple imaging modalities. While the cross-sectional studies cannot establish causality, the convergence of structural imaging data across sleep duration, regularity, and quality domains, summarized in Figure 1, provides a basis for further longitudinal and experimental investigation into the relationship between insufficient sleep and brain structure. The population-level nature of the ABCD study findings suggests that these associations are not limited to clinical sleep disorders, but extend to the wide range of sleep durations within developing adolescent populations, underscoring the public health relevance of further research into adolescent insufficient sleep.

## 5. Limitations and Future Directions

### 5.1. Cross-Sectional Evidence and Causality

Many neuroimaging associations (sleep duration correlating with cortical thickness, white matter integrity, gray matter volume) derive from cross-sectional studies that cannot establish temporal precedence or rule out reverse causation. Adolescents with pre-existing differences in brain structure might sleep differently due to neurobiological factors, rather than brain differences resulting from sleep patterns. Longitudinal studies with repeated imaging are sparse and typically limited to 1–3 year follow-up periods, which is insufficient to capture long-term developmental trajectories. Experimental sleep restriction studies in adolescents face ethical constraints limiting duration and severity of manipulation, leaving the effects of chronic real-world sleep patterns incompletely characterized. Intervention trials demonstrating that sleep improvements produce measurable changes in brain development, cognitive function, or mental health outcomes also remain limited.

### 5.2. Methodological Advances

Current sleep assessment methods have limitations. Polysomnography is expensive; actigraphy provides objective data but limited architectural information; self-report is subject to bias. Advances in wearable technology, smartphone-based assessment, and novel biomarkers may enable more feasible, scalable, and comprehensive sleep assessment. Research validating new assessment approaches and examining how to integrate them into research and clinical practice will enhance field progress.

Similarly, neuroimaging methods continue to evolve. Advances in MRI technology, analysis approaches, and multimodal integration may provide deeper insight into how sleep affects brain development. Studies should leverage emerging methods while maintaining rigorous validation standards.

### 5.3. Clinical and Translational Considerations

Several limitations constrain direct translation of research evidence to clinical recommendations. Optimal screening instruments feasible for primary care settings require validation, and economic analyses of screening and intervention programs are needed to inform resource allocation decisions. While acknowledging these limitations, clinicians might consider: (1) incorporating basic sleep screening into adolescent preventive care; (2) educating families about the biological basis of adolescent sleep needs and circadian changes; (3) providing targeted guidance on screen time and sleep hygiene when appropriate; (4) supporting school start time delays through professional advocacy when opportunities arise; and (5) assessing for sleep problems when evaluating adolescents presenting with cognitive complaints, academic difficulties, or mood symptoms. These approaches align with existing professional guidelines while remaining aware of implementation challenges and the need for ongoing evaluation of clinical effectiveness and cost-effectiveness in real-world settings.

### 5.4. Social Determinants and Implementation Barriers

Social determinants including family work schedules, transportation constraints, housing conditions, and neighborhood safety impact adolescent sleep but may be challenging to address through clinical interventions alone. Effective sleep health promotion likely requires coordination across healthcare, educational, and community systems, though models for such integrated approaches are still emerging. Targeted interventions addressing screen time and sleep hygiene represent feasible approaches at individual and family levels, while delayed school start times remain a modifiable, policy-level lever supported by the evidence reviewed above.

## 6. Conclusions

Insufficient sleep represents an important yet underrecognized public health problem with profound consequences for adolescent development. The evidence synthesized in this review suggests that insufficient sleep during adolescence is associated with a biologically rooted developmental mismatch between pubertal changes in sleep-wake timing and the demands of modern society, with consequences extending to detectable differences in brain morphology, including reduced white matter integrity, thinner cortical structure in frontal, parietal, and temporal regions, and smaller gray matter volumes in regions critical for cognitive and affective processing.

The convergence of these associations across multiple imaging modalities, sleep dimensions (duration, regularity, and timing), and study designs, including population-level findings from the ABCD Study, provides a consistent pattern of associations between insufficient sleep and measurable differences in the developing adolescent brain. The identification of the medial prefrontal cortex as a region where sleep, brain structure, and academic performance converge further highlights the functional significance of these structural associations. Recognizing sleep as a fundamental pillar of adolescent health, rather than a discretionary behavior, positions sleep health promotion as an essential component of comprehensive adolescent healthcare and public health policy.

## Figures and Tables

**Table 1:** Summary of structural neuroimaging studies linking sleep to brain morphology in adolescents and comparison with adult populations. GM = gray matter; WM = white matter; OFC = orbitofrontal cortex; PFC = prefrontal cortex; ACC = anterior cingulate cortex; mPFC = medial prefrontal cortex; PCC = posterior cingulate cortex; SLF = superior longitudinal fasciculus; DTI = diffusion tensor imaging; FA = fractional anisotropy; VBM = voxel-based morphometry.

Study	Design	Sample	Modality	Sleep Variable	Key Finding
*Sleep Duration*					
Jalbrzikowski et al. [31]	Cross-sectional	Adolescents, N = 225	Structural MRI: cortical thickness and subcortical volume; wrist actigraphy	Sleep duration, sleep timing, continuity, and regularity	Shorter sleep duration, later sleep timing, and poorer sleep continuity were associated with reduced cortical thickness and altered subcortical volumes across regions involved in sensory, cognitive, and emotional processing.
Yang et al. [32]	Longitudinal, observational cohort study	Adolescents, N = 8323	Resting-state fMRI, structural MRI (gray matter volume), behavioral/cognitive measures	Sleep duration	Chronic insufficient sleep during childhood is associated with persistent behavioral and neurobiological alterations, with disruptions in cortico-basal ganglia circuits and anterior temporal lobe structure potentially mediating effects on mental health and cognitive function
Spira et al. [33]	Longitudinal	Adults, N = 122, mean age: 66.6 years	Structural MRI (serial cortical thickness measurements	Sleep duration	Both short and long duration associated with accelerated cortical thinning in frontal and temporal regions (adult comparison)
Zitser et al. [34]	Longitudinal (28-years)	Adults, N = 613, baseline age: 42.3 ± 5.0 years	Structural MRI (VBM gray matter volume), Diffusion MRI (FA, RD), cognitive testing	Sleep duration	No differences in GM volume, WM microstructure, or cognition across trajectory groups (null)
*Sleep Regularity*					
Urrila et al. [35]	Cross-sectional	Adolescents, N = 177, mean age 14	Structural MRI (T1-weighted MRI, voxel-based morphometry for gray matter volume), academic performance	Weekday time in bed, weekend bedtime, weekend wake-up time	Shorter weekday sleep and later weekend sleep timing were associated with smaller gray matter volumes in frontal, anterior cingulate, and precuneus regions. Later weekend bedtime was linked to poorer school performance, with medial prefrontal/anterior cingulate cortex volume showing the strongest relationship with both sleep habits and academic outcomes.
*Sleep Quality*					
Sung et al. [36]	Cross-sectional	Adolescents, N = 56, ages 12–17	T1-weighted MRI, voxel-based morphometry with DARTEL for gray matter volume	Sleep disturbance severity measured by PSQI	Greater sleep disturbances were associated with reduced GM volume in frontal, OFC, insula, and other network regions.
Sexton et al. [37]	Longitudinal	Adults, N = 147, mean age 53.9	Structural MRI (cortical and hippocampal volume)	Sleep quality measured by PSQI	Poor sleep quality was associated with reduced frontal cortical volume and faster widespread cortical atrophy
Grau-Rivera et al. [38]	Cross-sectional	Adults, N = 1683, middle-aged (MRI: T1 N = 366; diffusion MRI N = 334)	Structural MRI, diffusion MRI, cognitive testing	Insomnia diagnosis	Insomnia was associated with poorer executive function, reduced GM volume in AD-related regions, and decreased white matter integrity
Neumann et al. [39]	Cross-sectional	Young adults, N = 1074, ages 22–35	Structural MRI (VBM)	Sleep quality measured by PSQI	Poor sleep quality was associated with altered GM volume in females, including hippocampal/parahippocampal differences; no significant effects were found in males.
*White Matter Microstructure*					
Telzer et al. [3]	Longitudinal	Adolescents, N = 48 (mean age 14.8 years at first sleep assessment; DTI scan at 16.3 years)	Diffusion MRI	Sleep variability, average sleep duration, bedtime variability	Greater variability in sleep duration was associated with lower white matter integrity (FA) across frontocortical, frontostriatal, and interhemispheric tracts
McMahon et al. [40]	Cross-sectional	Adults, N = 103, ages 18–90	DTI (FA)	Rest-activity rhythm stability	Greater stability associated with higher FA in corpus callosum and anterior corona radiata; not moderated by age group
Khalsa et al. [41]	Cross-sectional	Young adults, N = 33, ages 23–29	DTI (FA)	Sleep duration measured by PSQI	Shorter duration associated with reduced FA in orbitofrontal region and superior corona radiata (young adult comparison)
Reyes et al. [42]	Cross-sectional	Young adults, N = 73, mean age 22.5	DTI (FA)	Sleep duration measured by actigraphy	Long sleep duration was associated with lower white matter integrity (lower FA, higher radial diffusivity) in sleep-related tracts including the corpus callosum, corona radiata, and thalamic pathways.
*Population-Level Evidence*					
Cheng et al. [17]	Cross-sectional	N = 11,067, ages 9–11	Structural volumetrics, cognitive and psychiatric assessments	Sleep duration	Shorter duration associated with smaller volumes in OFC, PFC, temporal cortex, precuneus, and supramarginal gyrus; brain volume partially mediated link to psychopathology

## Data Availability

No new data were created in this study. Data sharing is not applicable to this article as no datasets were generated or analyzed during the current study.
